# ArsR Family Regulator MSMEG_6762 Mediates the Programmed Cell Death by Regulating the Expression of HNH Nuclease in Mycobacteria

**DOI:** 10.3390/microorganisms10081535

**Published:** 2022-07-29

**Authors:** Xiangke Duan, Xue Huang, Junqi Xu, Xue Li, Jingjing Niu, Xiaoli Du, Xiaoyu Wang, Jiang Li, Michael Kelly, Jiaohan Guo, Ke Zhang, Yu Huang, Biao Kan, Jianping Xie

**Affiliations:** 1 School of Life Sciences, Southwest University, Chongqing 400715, China; duanxke@hotmail.com (X.D.); 20192601790@cqu.edu.cn (X.H.); jj20200901@swu.edu.cn (J.X.); xueli931024@126.com (X.L.); jingjingniu0613@163.com (J.N.); 20181901038@cqu.edu.cn (X.W.); leemn1356@163.com (J.L.); jiaohanguo@gmail.com (J.G.); kzhang2016@foxmail.com (K.Z.); 18375633177@163.com (Y.H.); 2 Shenzhen Center for Disease Control and Prevention, Shenzhen 518055, China; 3 National Institute for Communicable Disease Control and Prevention, Chinese Center for Disease Control and Prevention, Beijing 102206, China; duxiaoli@icdc.cn (X.D.); kanbiao@icdc.cn (B.K.); 4 Saint John’s University, 2850 Abbey Plaza, Collegeville, MN 56321, USA; michael.kelly42113@gmail.com

**Keywords:** programmed cell death (PCD), *Mycobacterium smegmatis*, ArsR family regulator, HNH nuclease, DNA damage

## Abstract

Programmed cell death (PCD) is the result of an intracellular program and is accomplished by a regulated process in both prokaryotic and eukaryotic organisms. Here, we report a programed cell death process in *Mycobacterium smegmatis*, an Actinobacteria species which involves a transcription factor and a DNase of the HNH family. We found that over-expression of an ArsR family member of the transcription factor, *MSMEG_6762,* leads to cell death. Transcriptome analysis revealed an increase in the genes’ transcripts involved in DNA repair and homologous recombination, and in three members of HNH family DNases. Knockout of one of the DNase genes, *MSMEG_1275*, alleviated cell death and its over-expression of programmed cell death. Purified MSMEG_1275 cleaved the *M. smegmatis* DNA at multiple sites. Overall, our results indicate that the MSMEG_6762 affects cell death and is mediated, at least partially, by activation of the HNH nuclease expression under a stress condition.

## 1. Introduction

Programmed cell death (PCD) refers to a genetically regulated process that leads to cell suicide [1]. It is an essential mechanism in the development and homeostasis of multicellular organisms, and is beneficial to bacterial populations and genomes [2]. Recently, PCD systems have also been found in eubacteria, which play a key role in the survival of the population under environmental stresses, such as nutrients deprivation and antibiotics treatments [2,3,4].

A toxin–antitoxin (TA) system has been studied extensively as a mechanism for bacterial PCD [5,6]. TA systems usually consist of two genes encoding a toxin and an antitoxin that counteracts the lethal action of toxin [7,8,9]. A well-characterized chromosomal TA system involved in bacterial PCD is MazEF of *Escherichia coli* [10,11]. MazF, the toxin, is a sequence-specific endoribonuclease that cleaves mRNAs at ACA or ACU sites in *E. coli* [12]. The cleavage of mRNAs blocks protein synthesis for metabolism and survival and halts cell proliferation. YihE Kinase was identified as a central regulator of bacterial cell death mediated by the MazEF [13]. Several investigations have revealed that some stress conditions trigger the mazEF PCD system, including starvation [14,15], antibiotics [16,17], high temperature [17], DNA damage [17,18], and oxidative stress [17]. However, the MazEF-mediated PCD in *E. coli* was controversial due to its reproducibility [19,20,21]. PezAT, a member of type II TA system, and the toxin protein PezT can phosphorylate the ubiquitous peptidoglycan precursor uridine diphosphate-N-acetylglucosamine (UNAG), which inhibits the peptidoglycan synthesis and leads to cell death eventually [22]. Programmed bacterial deaths could also be induced by a variety of restriction–modification (RM) systems. RM systems commonly contain a modification enzyme capable of methylating specific DNA sequences in genomes and a restriction endonuclease capable of cleaving DNA lacking those methylations [23]. The PCD was also found in the sporulating bacteria, *Bacillus subtilis*. Under nutrient-limited conditions, the spore formation-related regulatory protein Spo0A regulates the sporulating killing factor skfA-H and sporulating delay protein sdpABC operons, which decided the fate of these bacteria—to live or to die [24,25].

*MSMEG_6762* is an ArsR family transcriptional regulator abundant in mycobacteria and other bacterial species, such as *Staphylococcus aureus*, *Shigella sonnei*, *Weissela cibaria*, and *Klebsiella pneumoniae*. The N-terminus of the ArsR family of transcription regulators contains a DNA-binding domain, which binds downstream promoters of target genes to regulate transcription [26]. Generally, ArsR family transcriptional regulators act as metal sensors and modulate the transcription of genes related to metal ion stress [26,27]. Additionally, some ArsR-type regulators, such as *HlyU* [28,29], *SloR* [30] and *PagR* [31], are involved in bacterial pathogenesis.

In this work, we found that the overexpression of MSMEG_6762 leads to cell death. MSMEG_6762 regulates the expression of HNH nuclease *MSMEG_1275*, which degrades DNA and eventually causes cell death. Knocking out *MSMEG_1275* relieved the bactericidal activity of MSMEG_6762. The study found a new PCD in *M. smegmatis*, which is associated with an ArsR family regulator and HNH nuclease cascade, and which constitutes a live-or-die response decision.

## 2. Materials and Methods

### 2.1. Bacterial Strains, Plasmids and Growth Conditions

The *M. smegmatis* and *E. coli* strains and plasmids used in this study are shown in Table S1. The *E. coli* strain DH5α was used for cloning. *E. coli* strains were grown on LB broth agar or in LB broth, 37 °C, 200 rpm. *M. smegmatis* mc^2^ 155 was grown in 7H9 liquid medium (Difco) supplemented with 0.05% *w*/*v* Tween 80, 0.5% glycerol, and 0.5% glucose or was grown on 7H10 agar supplemented with 1% glycerol and 0.5% glucose. Restriction enzymes, T4 DNA ligases, and DNA polymerases were purchased from Takara. Ampicillin, kanamycin, hygromycin were bought from Sangon Biotech Co., whose stock solutions were freshly prepared and filter sterilized. When required, the following antibiotics were used at the final concentration: ampicillin, 100 μg/mL; kanamycin, 500 μg/mL for *E. coli* or 200 μg/mL for *M. smegmatis*; hygromycin, 50 μg/mL. All cultures were incubated at 37 °C.

### 2.2. Plasmids and Expression Strains Construction

Mycobacterial expression vector pALACE used in this study has been described previously [32], and all primers used in this study are shown in Supplemental Table S2. The coding sequence of *MSMEG_6760* was amplified with primer pair 6760-F/-R, the coding sequence of *MSMEG_6762* was amplified with primer pair 6762-F/R, and the coding sequence of *MSMEG_6760-MSMEG_6762* was amplified with primer pair 6760-6762-F/-R. All were cloned as *Bam*H I/*Eco*R I fragments into correspondingly digested pALACE to form pALACE-6760, pALACE-6762 and pALACE-6760-6762. *MSMEG_5583*, *MSMEG_1756*, *MSMEG_5876*, *MSMEG_3404*, *MSMEG_1275*, and *MSMEG_2148* were amplified with their own primers, and subsequently cloned as *Afl* II/*Nde* I fragments into correspondingly digested pALACE to form pALACE-5583, pALACE-1756, pALACE-5876, pALACE-3404, pALACE-1275, and pALACE-2148, respectively. These plasmids were then electroporated into *M. smegmatis* mc^2^ 155 to generate overexpression strains, respectively.

### 2.3. Effect of Conditional Expression of Genes on Mycobacterial Growth and Viability

Both solid medium and liquid culture were used to test the toxicity of target genes in *M. smegmatis*. To assay the effect of expression on solid medium, strains were grown in 7H9 media to an OD_600_ of approximately 1.0. Each strain was streaked onto an agar plate supplemented with hygromycin, with or without 1% acetamide to induce expression of either the target genes. After 3 days of growth at 37 °C, pictures of the plates were taken by an image analysis system (Furi science & technology Co., Ltd., Shanghai, China). For toxicity assessment in liquid culture, strains were grown in 7H9 media to an OD_600_ = 0.1 as the expression was induced with 1% acetamide, with OD_600_ and CFU measured over time. Each experiment was performed in triplicate at each time point.

For the Western blot detection of His-tagged MSMEG_6762 and MSMEG_6760, bacterial pellets were harvested and disrupted by ultrasonication. Samples were subjected to SDS-PAGE, and the His-tagged proteins were detected by the mouse anti-His antibody (TIANGEN, Beijing, China).

### 2.4. Site-Directed Mutagenesis

The sequences of all primers used in site-directed mutagenesis are shown in Table S2. Four conserved sites were introduced into the *MSMEG_6762* by site-directed mutagenesis [33] with the recombinant vectors T-*MSMEG_6762* isolated from *E. coli* DH5α as a template. The mutagenic primers are shown in Table S2. Mutation of 18L (CTC) to A (GTA) used primer pair 6762 _L18A_-F/-R, 24R (AGG) to A (GTA) used primer pair 6762 _R24A_-F/-R, 54H (CAT) to A (GTA) used primer pair 6762 _H54A,_ and 58 L (CTC) to A (GTA) used primer pair 6762 _L58A_-F/-R, respectively. The mutations were confirmed by DNA sequencing using primers 6762-F and 6762-R. For MSMEG_1275, mutation of 258H (CAT) to A (GTA) used primer pair 1275 _H258A_-F/-R, 272N (AAC) to A (GTA) used primer pair 1275 _N272A_-F/-R, and 281N (AAC) to A (GTA) used primer pair 1275 _N281A_-F/-R, respectively.

### 2.5. RNA-Seq Analysis

For RNA-Seq analysis, MS-VEC and MS-6762 were grown to a turbidity of 0.4, and then the final concentration of 1% acetamide was added to induce MSMEG_6762. After induction for 9 h, cells were harvested. The total amount of RNA was extracted using RNeasy Mini Kit (Qiagen, GmBH, Hilden, Germany) following the manufacturer’s instructions and was checked for a RIN number to inspect RNA integrity by an Agilent Bioanalyzer 2100 (Agilent technologies, Santa Clara, CA, USA). Qualified RNA from the previous steps was further purified by Rneasy micro kit (Qiagen, GmBH, Hilden, Germany) and Rnase-Free Dnase Set (Qiagen, GmBH, Hilden, Germany). RNA-Seq was performed by Shanghai Biochip Inc. Results were analyzed in edgeR [34] with Significance Analysis of Microarrays considered significant at *q* < 0.05.

### 2.6. Terminal Deoxynucleotidyl Transferase dUTP Nick end Labeling (TUNEL) Assay

Stationary cultures of MS-VEC, MS-6762 and MS-1275 were reinoculated into 7H9 medium, and acetamide was added into the cultures while the OD_600_ reached 0.1. Aliquots of mycobacterial cells were collected from *M. smegmatis* cultures induced for 8 h, and the TUNEL assay was performed according to the in situ cell death detection kit (Roche Diagnostics, Indianapolis, IN, USA) instruction. Samples were analyzed by FACS; the FITC signal was analyzed with an emitting laser at 488 nm and bandpass filter of 525/15 nm using a BD Aria II flow cytometer (BD Biosciences, San Jose, CA, USA) with a 70-μm nozzle. For each sample, 10,000 events were acquired, with TUNEL staining gradations expressed as percentages of total gated cells.

### 2.7. Protein Expression and Purification

The sequences of all primers used in protein expression and purification are shown in Table S2. Recombinant MSMEG_6762 and MSMEG_1275 were expressed in *E. coli* according to a published protocol [35]. Briefly, the MSMEG_6762 and MSMEG_1275 coding region were amplified by PCR from the genomic DNA of *M. smegmatis* using pET6762F and pET6762R, or pET1275F and pET1275R. The gene was cloned into pET28a expression vector, *E. coli* BL21 cells carrying recombinant plasmids were induced with 1 mM IPTG (isopropyl β-D-thiogalactopyranoside), and the bacteria were incubated for 4 h, at 37 °C. Cell lysates were prepared by sonication, and His-MSMEG_6762 was purified by binding to Ni-NTA (GenScript, Tokyo, Japan) equilibrated with wash Buffer (50 mM NaH_2_PO_4_, 300 mM NaCl, 20 mM imidazole, pH 8.0), and eluted into the same buffer but containing 250 mM imidazole. His-MSMEG_1275 was dissolved in Buffer A (100 mM NaH_2_PO_4_, 300 mM NaCl, 9 M Urea, 5 mM imidazole, 10mMTris-HCl, 1 mM β-mercaptoethanol, pH 7.4). His-MSMEG_1275 was purified by binding to Ni-NTA (GenScript, Nanjing, China) equilibrated with wash Buffer (100 mM NaH_2_PO_4_, 300 mM NaCl, 9 M Urea, 20 mM imidazole, 10 mM Tris-HCl, 1 mM β-mercaptoethanol, pH 7.4) and eluted into the same buffer but containing 250 mM imidazole. The elution fractions containing His-MSMEG_1275 were diluted in Buffer B (100 mM NaH_2_PO_4_, 300 mM NaCl, 0.1 mM EDTA, 0.01% Triton X-100, 10 mM Tris-HCl, 20% Glycerol, pH 7.4), and concentrated by Millipore Amicon^®^ Ultra-4. Protein concentration was detected by Bicinchoninic Acid (BCA) Assay (TIANGEN, Shanghai, China).

### 2.8. DNA Digestion Assay

The digestion assays were based on those used by Moodley et al. [36] with some modification. Briefly, the assays were performed with 10 μg/mL of MSMEG_1275 and 6.25 μg/mL *M. smegmatis* genome DNA. Digestion experiments were conducted in 10 mM HEPES, pH 7.0. Then, 5 mM β-mercaptoethanol was added and the final concentration of β-mercaptoethanol was 0.05 mM. Divalent metal ions (Ni^2+^, Mg^2+^, Zn^2+^, and Cu^2+^) were tested at concentrations of 1 mM as cofactors. After 5 h, 10 μL of sample was removed, and the reaction was stopped by addition of EDTA to a final concentration of 0.025 mM and DNA loading buffer (Takara, Kusatsu, Japan). Samples were analyzed by 1% agarose gel electrophoresis.

### 2.9. Electrophoretic Mobility Shift Assays (EMSA)

To evaluate the binding of His-MSMEG_6762 to the operator promoter regions, specific primers (Supplemental Table S2) were used to amplify the genomic DNA of *M. smegmatis*. DNA substrate and increasing concentrations of protein (as indicated in the legend of the corresponding figure) were incubated for 20 min in EMSA buffer (100 mM Tris-HCl, pH 8.0, 100 mM NaCl, 1 mM DTT and 10% glycerol), at room temperature. Products were separated on native 5% polyacrylamide gels (PAGE) in 0.5 × TBE buffer, at 4 °C, stained with GoldView, and visualized under UV-transmitting light.

### 2.10. Construction of Deletion Mutant Strains

The sequences of all primers used in knockout and overexpression are shown in Table S2. The genes of *M. smegmatis*mc^2^ 155 was disrupted using recombineering approach previously described [37]. The regions near the deletions were verified by PCR followed by DNA sequencing.

### 2.11. Statistical Analysis

Data from at least three biological replicates were used to calculate means and standard deviation (SD) for graphing purposes. Statistical analysis employed the unpaired Student’s *t* test, asterisks indicate statistically significant difference (* *p* < 0.05; ** *p* < 0.01; *** *p* < 0.001).

## 3. Results

### 3.1. Expression of MSMEG_6762 Causes the Cell Death of M. smegmatis

**Figure 1:**
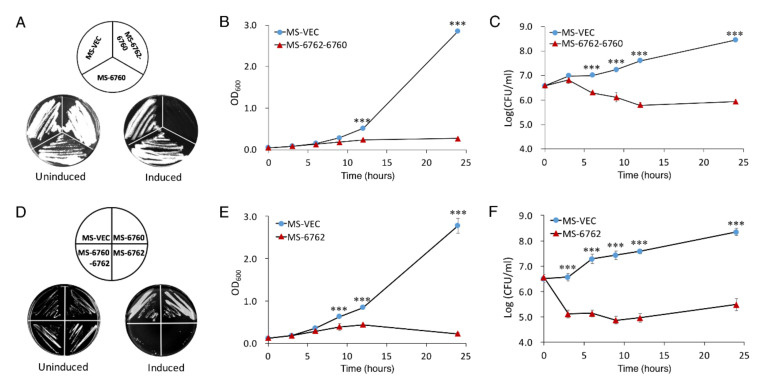
**Effect of MSMEG_6760 and MSMEG_6762 on the growth and viability of *M. smegmatis*.** Growth on 7H10 plates with 50 μg/mL hygromycin without (left) and with (right) 1% acetamide (**A**,**D**), were incubated for 3 days. *M. smegmatis* hosts containing pALACE-based constructs were cultured in 7H9 medium supplemented with 50 μg/μL hygromycin without (left) and with (added at OD_600_ = 0.1). Cell growth of MS-6762-6760 (**B**), MS-6762 (**E**) and viability (CFU/mL) of MS-6762-6760 (**C**), MS-6762 (**F**) were tested at indicated intervals. MS-VEC, *M. smegmatis* with pALACE plasmid. MS-6062, *M. smegmatis* with pALACE-*MSMEG_6760*-*MSMEG_6762* plasmid. MS-6762: with pALACE-*MSMEG_6762* plasmid. Experiments were performed in triplicate. Data are represented as mean +/− SEM. Significance of MS-VEC strain compared to MS-6762 strain was determined using a Student’s *t* test: *** *p* < 0.001.

*MSMEG_6762*-*MSMEG_6760* was predicted as a toxin–antitoxin pair system in *M. smegmatis* (https://db-mml.sjtu.edu.cn/TAfinder/index.php, accessed on 1 December 2013). MSMEG_6760 is a predicted toxin protein, and MSMEG_6762 is the predicted antitoxin protein [38,39]. In order to confirm this perdition, we performed a co-transcription analysis of *MSMEG_6762-MSMEG_6760*. As shown by RT-PCR, a single band of ~500 bp was detected using a forward primer that bound to *MSMEG_6760* and a reverse primer that bound to *MSMEG_6762* using cDNA synthesized from the total RNA as template, indicating that these two genes are co-transcribed. No bands were obtained using total RNA as a template (Figure S1). This result revealed that *MSMEG_6762*-*MSMEG_6760* are co-translated and form an operon. We then study this toxin–antitoxin pair module as in Frampton et al. [40]. The putative toxin gene was inserted into pALACE under the control of an acetamide-inducible promoter. To ensure the effect was observed due to the production of MSMEG_6760 alone, the operon *MSMEG_6762*-*MSMEG_6760* was also cloned in the same manner, thus preventing the effect of the toxin protein by co-expressing the cognate antitoxin. The empty vector pALACE (MS-VEC) was used as a control. However, in the presence of acetamide, cells expressing MSMEG_6760 were able to grow on a 7H10 agar plate, while cells co-expressing toxin and antitoxin failed to grow (Figure 1A, right panel). All three strains could grow normally on a 7H10 agar plate without acetamide (Figure 1A, left panel). A toxic effect of co-expressing MSMEG_6762-MSMEG_6760 was also exhibited in the liquid culture, as shown by the reduction in turbidity (OD_600_) and colony forming units (CFU) (Figure 1B,C). As shown in Figure 1A–C, this result was exactly the opposite of expectation. Since the expression of putative toxin protein MSMEG_6760 did not affect cell growth, should the putative antitoxin protein MSMEG_6762 be a potent toxin protein? To test this hypothesis, we cloned the region of *MSMEG_6762* into pALACE plasmid, and then transferred it into *M. smegmatis* host. In the presence of acetamide, cells expressing MSMEG_6762 could not grow on a 7H10 agar plate (Figure 1D) and exhibited a notable decrease in cell growth, as shown by the reduction in turbidity (OD_600_) and colony-forming units (CFU) (Figure 1E,F). The expression of MSMEG_6760 and MSMEG_6762 were verified by Western blot (Figure S2A,B). These results suggest that overexpression of MSMEG_6762 is lethal for *M. smegmatis*.

### 3.2. L18, R24, H54, and L58 Residues Are Critical for the Toxicity of MSMEG_6762

**Figure 2:**
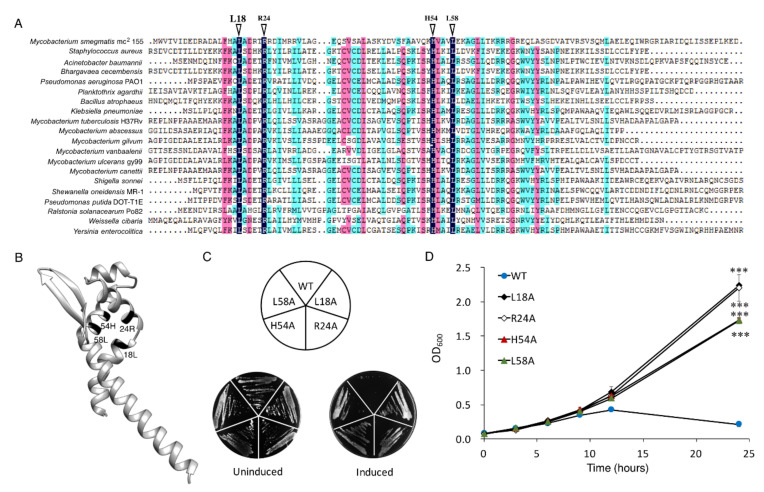
**Key residues for MSMEG_6762 toxicity.** (**A**) Conserved amino acid residues of *MSMEG_6762*. (**B**) Predicted 3D structure of *MSMEG_6762*. Toxicity results of single-site mutagenesis of 18L, 24R, 54H, and 58L of *MSMEG_6762* in solid (**C**) and liquid medium (**D**). MS-VEC: *M. smegmatis* with pALACE plasmid. MS-6762: with pALACE-*MSMEG_6762* plasmid. WT indicates the wild-type *MSMEG_6762* protein; the remainders are mutated proteins. The number in the mutated protein indicates the position of the amino acid in *MSMEG_6762*. Log-phase cultures were streaked on 50 μg/mL hygromycin 7H10 plates, with or without 1% acetamide. Experiments were performed in triplicate. Data are represented as mean +/− SEM. Significance of mutant strains compared to MS-6762 WT strain was determined using a Student’s *t* test: *** *p* < 0.001.

*MSMEG_6762* was identified as an ArsR transcriptional factor [41] with four amino acid residues conserved among the ArsR family regulator (Figure 2A). The 3D structure predicted by Phyre2 protein homology recognition engine [42] indicated that the conserved domain is situated at the end of one helix and R54, close to L58. The four amino acid residues might be crucial for DNA binding (Figure 2B). Then, we performed site-directed mutagenesis on L18, R24, H54, and L58 to explore the significance of the conserved residues in determining toxicity of MSMEG_6762. Substitution of any one of these amino acid residues with alanine abolished the toxicity of *MSMEG_6762*, both in liquid and solid medium (Figure 2C,D). The results indicate that residues L18, R24, H54, and L58 are critical in the toxicity of *MSMEG_6762*.

### 3.3. Overexpression of MSMEG_6762 Induces the DNA Damage in M. smegmatis

**Figure 3:**
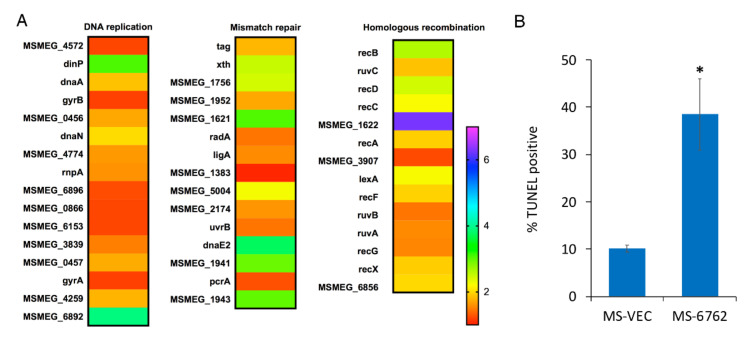
**DNA damage induced by *MSMEG_6762*.** (**A**) Heat maps of DNA repair related pathway. The relative fold change in expression level in several pathways was calculated and visualized over time using Excel; heat map was made with GraphPad Prism 6.0. (**B**) The percentage of TUNEL-positive of MS-VEC and MS-6762 (mean ± SD at 12 h after induction). Data are represented as mean +/− SEM. Significance of MS-6762 strain compared to MS-VEC strain was determined using a Student’s *t* test: * *p* < 0.05.

*MSMEG_6762* is a transcriptional regulator governing the expression of target genes [41]. To find the genes underlying the lethal effect of *MSMEG_6762*, RNA-Seq based transcriptome analysis was performed. Upon *MSMEG_6762* overexpression, at least 580 genes were upregulated, and 1127 genes were downregulated using log2 fold change (greater than 1 or less than 1) as a threshold (Table S3). The genes involved in mismatch repair, nucleotide excision repair, base excision repair, and homologous recombination were upregulated at least two-fold (log2 fold change greater than 1) (Table 1) (Figure 3A). Bacterial SOS is a global response to DNA damage to arrest the cell cycle, and initiate DNA repair. *RecA*-*lexA* modulates the SOS response. During normal growth, LexA encoded by the *lexA* gene acts as a repressor by binding to an operator DNA of a specific sequence, the SOS box, and prevents their expression [43,44,45]. Upon DNA damage, single-stranded DNA occurs [46]. RecA binds to these single-stranded regions and is converted to an active form to stimulate the self-cleavage of LexA [47]. The *recB*, *recC*, and *recD* gene-encoded proteins comprise a RecBCD complex, which is required for recombinational DNA repair of double-stranded DNA (dsDNA) breaks in bacteria [48,49]. The RuvA and RuvB proteins form a complex that catalyzes branch migration, and RuvC catalyzes resolution of Holliday junctions [50,51,52]. Real-time analyses were performed for selected three genes to confirm the RNA-seq results, including *MSMEG_1620* (the most highly upregulated gene), *lexA* and *recA* (two genes involved in DNA repair pathway). The real-time PCR results are as follows: up-regulated (5.11 times), up-regulated (4.27 times), and up-regulated (2.20 times) (Figure S3), which are in good agreement with the RNA-seq data. These data suggest that the expression of *MSMEG_6762* resulted in DNA damage in *M. smegmatis*.

To assess DNA double-strand break potentially caused by *MSMEG_6762* expression, we measured the DNA fragmentation in *M. smegmatis* by the terminal deoxynucleotidyltransferase-mediated dUTP-biotin nick end labeling (TUNEL) assay (Figure 3B). The percentage of cells with DNA breaks in *MSMEG_6762* overexpression strain reaches to 38% after 12 h induction, 3.82-fold higher than the MS-VEC strain. The data indicates that the expression of *MSMEG_6762* induces DNA damage in *M. smegmatis*.

### 3.4. MSMEG_6762 Causes Cell Death by an Unregulated HNH Nuclease MSMEG_1275

**Figure 4:**
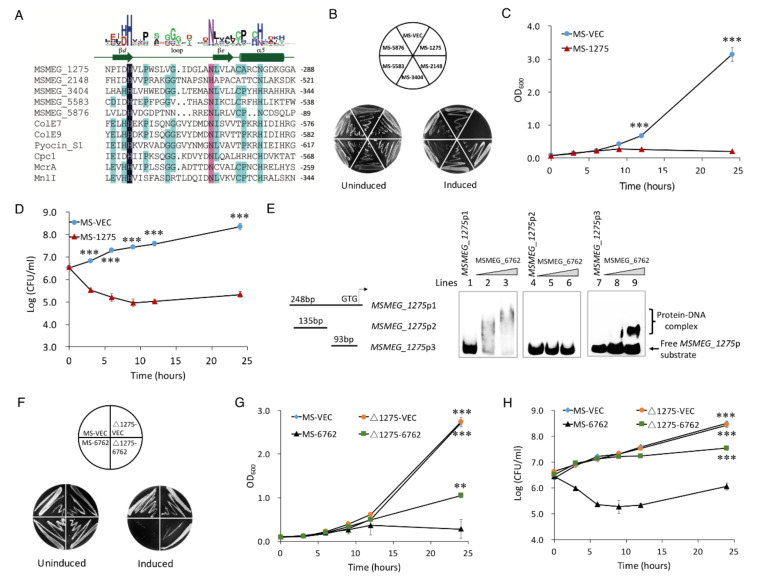
***MSMEG_6762* causes cell death by an unregulated HNH nuclease MSMEG_1275.** (**A**) Sequence alignment of HNH proteins. (**B**) Overexpression of HNH domain genes on 7H10 plates. (**C**) The effect of *MSMEG_1275* on the cell growth of MS-1275. (**D**) The bactericidal activity of HNH nuclease MSMEG_1275 on cell viability of MS-1275. Log-phase cultures were streaked on 50 μg/mL hygromycin 7H10 plates with or without 1% acetamide. Significance of MS-VEC strain compared to MS-1275 strain was determined using a Student’s *t* test: *** *p* < 0.001. (**E**) EMSA assays for the binding of *MSMEG_6762* to *MSMEG_1275* promoter DNA fragments. The *MSMEG_1275* promoter DNA substrates were co-incubated with gradually increasing concentrations of MSMEG_6762 protein (0, 2 and 4 μM). (**F**) The effect of HNH nuclease MSMEG_1275 on *MSMEG_6762* mediated cell death in solid culture medium. Log-phase cultures were streaked on 50 μg/mL hygromycin 7H10 plates with or without 1% acetamide. (**G**,**H**) The effect of HNH nuclease MSMEG_1275 on *MSMEG_6762* mediated cell death in liquid culture medium. Experiments were performed in triplicate. Data are represented as mean +/− SEM. Significance of tested strains compared to MS-6762 strain was determined using a Student’s *t* test: ** *p* < 0.01 and *** *p* < 0.001.

HNH motif is a small DNA binding and cleavage module characterized by two tightly conserved histidine residues separated by an asparagine residue [53]. To date, more than 1000 HNH motif-containing proteins have been identified from bacteria, archaea, and eukaryotes [54]. The largest subgroup of HNH motif-containing proteins with known function is the site-specific homing endonucleases [55], such as Cpc [56], I-TevIII [57], and I-BasI [58]. Bacterial toxins with HNH motifs include E7 [59], E9 [60], colicins, and pyocins S1, S2 [61]. Both E7 and E9 are endonucleases active on single- and double-stranded DNA, but with no clear specificity, and result in cell death [62,63]. Pyocin S1 and S2 exhibit DNase activity, which can degrade cellular DNA in susceptible cells [61]. HNH motif has also been identified in restriction or repair enzymes, such as MnlI [64] and McrA [65]. RNA-Seq was used to explore the relationship between *MSMEG_6762* expression and DNA damage. The data showed that five HNH motif-containing proteins were upregulated during the expression of *MSMEG_6762*, including *MSMEG_5583*, *MSMEG_5876*, *MSMEG_3404*, *MSMEG_1275*, and *MSMEG_2148* (Table 2 and Figure 4A). To the extent that *MSMEG_6762* is a transcriptional factor, HNH motif-containing proteins might cause such DNA damage. To test this hypothesis, we overexpressed these five genes in *M. smegmatis* solely. The result shows that only overexpression of *MSMEG_1275* greatly inhibited cell growth both on 7H10 agar (Figure 4B) and 7H9 liquid culture medium (Figure 4C).

Since *MSMEG_1275* was upregulated in response to the expression of *MSMEG_6762*, the expression level of *MSMEG_1275* might be regulated by *MSMEG_6762* directly. To test whether recombinant *MSMEG_6762* (Figure S4A) can interact with *MSMEG_1275* promoter, an EMSA assay was performed. As shown in Figure 4E (lines 1–4), when the MSMEG_1275p1 DNA substrate (100 bp) was co-incubated with increasing concentrations of recombinant His-MSMEG_6762 (0, 2, and 4 μM, respectively), clear shifted bands were observed. *MSMEG_6762* specifically bound to the MSMEG_1275p2 DNA substrate (Figure 4, lines 5–8); however, no band shifted by the shorter MSMEG_1275p3 DNA substrate (Figure 4E, lines 9–12). This result indicates that *MSMEG_6762* can specifically bind to the *MSMEG_1275* promoter region.

To confirm *MSMEG_1275* is the downstream regulation target of *MSMEG_6762*, we knocked out *MSMEG_1275* and expressed *MSMEG_6762* in the Δ*MSMEG_1275* strain. If *MSMEG_1275* is the downstream regulation target of *MSMEG_6762*, the lethal effect of *MSMEG_6762* will be abolished or relieved. As we expected, knockout *MSMEG_1275* relieved the bactericidal activity of *MSMEG_6762* on a 7H10 agar plate, as well as in the liquid culture medium (the OD_600_ of Δ1275-6762 reached 0.946 after 24 h induction) (Figure 4F, G). Knockout *MSMEG_1275* aborted the bacteria killing activity of *MSMEG_6762*, but caused a delay in growth (Figure 4H). These results demonstrate that *MSMEG_1275* is the downstream target of *MSMEG_6762*.

### 3.5. MSMEG_1275 Mediates Double-Stranded Digestion of M. smegmatis Chromosome DNA

**Figure 5:**
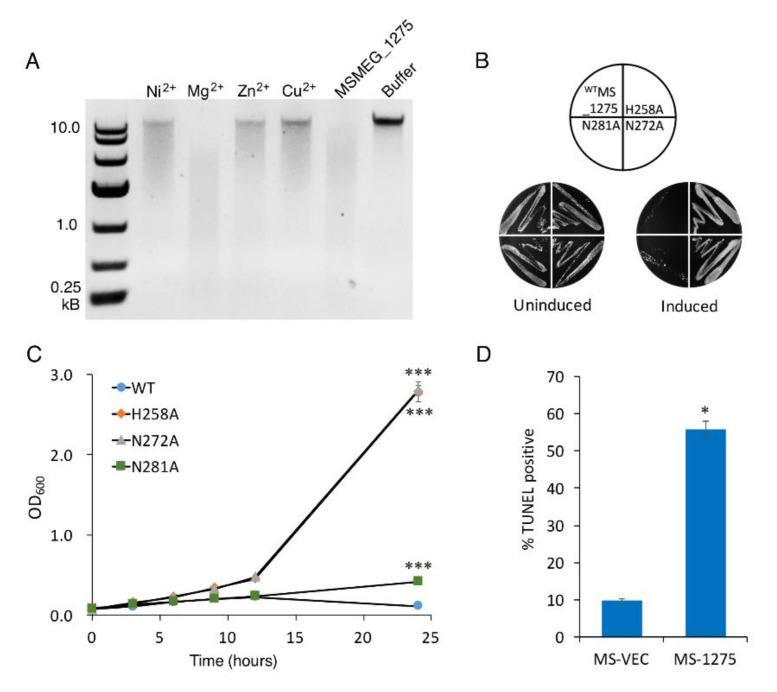
**MSMEG_1275 is an HNH nuclease and cleaves double-stranded DNA of *M. smegmatis*.** (**A**) Effect of divalent cations on the DNase activity. Toxicity results of single-site mutagenesis of 258H, 272N and 281N of MSMEG_1275 in solid (**B**) and liquid medium (**C**). (**D**) The percentage of TUNEL-positive of MS-VEC and MS-1275 after 12 h induction. Data are represented as mean +/− SEM. Significance of mutant strains compared to MS-1275 WT strain was determined using a Student’s *t* test: * *p* < 0.05 and *** *p* < 0.001.

To determine whether MSMEG_1275 possesses nuclease activity, we examined its ability to cleave the *M. smegmatis* chromosome. Purified MSMEG_1275 (Figure S4B) was tested for nuclease activity in the presence of a variety of divalent metal ions. Without metal ions, MSMEG_1275 cleaves the chromosome at multiple sites, as evidenced by the formation of a continuum of DNA fragments with varying lengths (Figure 5A). The enzyme exhibited high activity with 1 mM Mg^2+^ in the reaction buffer, less activity with 1 mM Ni^2+^, Zn^2+^, and very low activity with 1 mM Cu^2+^ (Figure 5A). In the second subfamily of HNH superfamily, the second His residue is usually substituted by a conserved Asn residue and forms a HNN motif [66]. To check whether these conserved residues constitute the HNN motif, we constructed three MSMEG_1275 mutants, H258A, N272A and N281A, and we found that the mutation of H258 or N272 abolished the MSMEG_1275 bacteria-killing activity both in liquid and solid medium in vivo, and mutation of N281 decreased the activity of MSMEG_1275 (the OD_600_ of N281A reached 0.417 after 24 induction) (Figure 5B,C). These results indicate that MSMEG_1275 has nuclease activity, and the conserved residues H258 and N272 are essential for its nuclease activity. Next, we measured the DNA fragmentation in *M. smegmatis* by the TUNEL assay. The results showed that the percentage of cells with DNA breaks in MSMEG_1275 overexpression strain reached 55%, 5.72-fold higher than the MS-VEC strain (Figure 5D). The data indicates that the HNH nuclease MSMEG_1275 cleaves *M. smegmatis* chromosome DNA.

## 4. Discussion

PCD in bacteria is a form of active suicide phenomenon that is controlled by related genes. Within the group of bacteria, a part of these bacteria that has programmed cell death is important to the entire bacterial population. Here, we report for the first time that the ArsR family transcriptional regulator *MSMEG_6762* is involved in programmed cell death. We demonstrated that the overexpression of *MSMEG_6762* causes bacterial death in *M. smegmatis* cells and identified that residues L18, R24, H54, and L58 are crucial for its activity. We further showed that *MSMEG_6762* regulates the expression of HNH nuclease *MSMEG_1275*, which cleaves the double-stranded DNA in an iron-independent manner and eventually causes bacterial cell death. Moreover, we found that mutation of H258 or N272 aborted the nuclease activity of *MSMEG_1275*. Together, our findings unveiled a novel programmed cell death pathway in *M. smegmatis* which is consistent with an ArsR family transcription factor, *MSMEG_6762*, and an HNH nuclease, *MSMEG_1275*. Upon the treatment of amikacin, *MSMEG_6762* was expressed and binds the promoter of *MSMEG_1275*, and then MSMEG_1275 degrades the DNA of the host, which leads to cell death.

Zhang et al. [67] and Gao et al. [41] conducted a similar overexpression experiment of *MSMEG_6762* in *M. smegmatis*, but no bacteria-killing activity of MSMEG_6762 was documented, which might be related to the plasmid and promoters used in the different expression vectors in *M. smegmatis*. Zhang et al. [67] found that the overexpression of MSMEG_6760 and MSMEG_6762-6760 did not affect the growth of *M. smegmatis*. However, the expression of target proteins has not been confirmed by Western blot. In this study, we assessed the expression level of MSMEG_6762 and MSMEG_6760 by Western blot (Figure S1) and confirmed that overexpression of MSMEG_6762 causes cell death in *M. smegmatis*. We also tested the toxicity of MSMEG_6762 in another mycobacteria expression, plasmid pNIT [68], overexpression of MSMEG_6762 by pNIT was toxic to *M. smegmatis* as well (Figure S5). Hence, we confirm that the expression of MSMEG_6762 was toxic to *M. smegmatis*.

The ArsR family transcriptional regulators have been found to be involved in various important cellular events, such as metal ion homeostasis, biofilm formation, and virulence [69]. phoPR is one of the two-component systems in mycobacteria and plays an important role in cell wall biosynthesis [70], virulence, hypoxic response [71], and pH response [72]. The expression of *phoP* was positive regulated by MSMEG_6762 [41]. There is a zero-fold change in information in our transcriptome data. *MSMEG_3932* (*hspX*), encoding a small heat shock protein in *M. smegmatis,* also positively regulated by MSMEG_6762. However, *hspX* was the most down-regulated gene upon MSMEG_6762 overexpression in our study. Thus, we believe that *phoP* and *hspX* might not be involved in MSMEG_6762-mediated cell death in *M. smegmatis*. Since the expression of MSMEG_6762 resulted in cell death in *M. smegmatis*, this means *MSMEG_6762* might be induced under lethal conditions, such as antibiotic, heat or heavy metal treatment. It is interesting to know the physiological role of MSMEG_6762 in *M. smegmatis*.

HNH motif is a small nucleic-acid binding and cleavage module, including site-specific homing endonucleases, non-specific endonuclease [73], DNA fragmentation during cell apoptosis [74] and repair of enzymes [75]. We overexpressed five HNH motif nucleases in this study and found only one was lethal for bacteria. HNH endonuclease/nuclease motif-containing proteins have been shown to play an important role in the competition between rival bacteria [76]. Furthermore, knockout *MSMEG_1275* only can abolish a part of the killing activity of *MSMEG_6762* (as the OD_600_ of Δ1275-6762 was 4.27-fold higher than MS-6762 after 24 h induction), which means there is more than one pathway involved in the *MSMEG_6762*-induced cell death. It would be interesting to isolate the surviving mutants and identify the possible pathways which are involved in *MSMEG_6762*-caused cell death.

In conclusion, we have shown that a novel PCD pathway has been identified in *M. smegmatis*. In this PCD pathway, *MSMEG_6762* works as a regulator, and MSMEG_1275 works as an executor which controls the cell fate of bacteria. This finding has expanded the understanding of ArsR family transcriptional regulators, because this is the first report of this family member serving as a death regulator in bacterial PCD.

## Figures and Tables

**Table 1:** Transcriptional profile of genes in the response to MEMSG_6762 expression reveals the DNA damage in *M. smegmatis*.

Gene	Description	FC ^a^	*p*-Value
*MSMEG_1620*	hypothetical protein	7.122	3.99 × 10^−25^
*MSMEG_1622*	DNA repair polymerase	6.262	9.78 × 10^−23^
*MSMEG_6892*	Replicative DNA helicase	3.898	7.00 × 10^−13^
*dnaE2*	Error-prone DNA polymerase	3.745	9.14 × 10^−11^
*MSMEG_1943*	ATP-dependent DNA helicase	2.996	1.34 × 10^−84^
*recB*	Exodeoxyribonuclease V subunit beta	2.623	6.01 × 10^−67^
*xth*	Exodeoxyribonuclease III	2.516	4.05 × 10^−62^
*recD*	Exodeoxyribonuclease V subunit alpha	2.462	2.13 × 10^−58^
*MSMEG_1756*	Endonuclease VIII	2.449	4.88 × 10^−41^
*lexA*	LexA repressor	2.242	5.94 × 10^−52^
*recC*	Exodeoxyribonuclease V subunit gamma	2.222	9.98 × 10^−51^
*dnaN*	DNA polymerase III subunit beta	2.006	3.25 × 10^−43^
*MSMEG_6856*	MmgE/PrpD family protein	1.994	5.01 × 10^−9^
*recF*	Recombination protein F	1.955	1.13 × 10^−40^
*recA*	Recombinase A	1.934	1.19 × 10^−40^
*ruvC*	Holliday junction resolvase	1.859	1.28 × 10^−36^
*tag*	DNA-3-methyladenine glycosylase I	1.803	7.12 × 10^−35^
*MSMEG_4259*	DNA polymerase III, epsilon subunit	1.778	5.43 × 10^−34^
*MSMEG_1952*	ATP-dependent DNA helicase	1.719	1.33 × 10^−32^
*MSMEG_2174*	Superfamily protein I DNA or RNA helicase	1.617	1.15 × 10^−29^
*ligA*	NAD-dependent DNA ligase LigA	1.565	7.19 × 10^−28^
*ruvA*	Holliday junction DNA helicase RuvA	1.550	1.91 × 10^−26^
*recG*	ATP-dependent DNA helicase RecG	1.528	5.29 × 10^−26^
*MSMEG_3839*	DNA polymerase I	1.488	7.53 × 10^−26^
*ruvB*	Holliday junction DNA helicase RuvB	1.437	1.18 × 10^−23^
*pcrA*	ATP-dependent DNA helicase PcrA	1.258	2.30 × 10^−19^
*MSMEG_6896*	Single-stranded DNA-binding protein	1.241	6.98 × 10^−19^
*MSMEG_4572*	DNA polymerase III, delta subunit	1.215	6.32 × 10^−18^
*MSMEG_6153*	DNA polymerase III subunit delta’	1.206	1.45 × 10^−17^
*MSMEG_1383*	Endonuclease IV	1.010	2.63 × 10^−13^

^a^ FC, log2 fold change.

**Table 2:** Upregulated HNH family genes in response to MEMSG_6762 expression in *M. smegmatis*.

Gene	Description	FC ^a^	*p*-Value
*MSMEG_5583*	HNH endonuclease	4.334	1.52 × 10^−14^
*MSMEG_5876*	H-N-H endonuclease F-TflIV	1.411	1.20 × 10^−17^
*MSMEG_3404*	HNH endonuclease domain-containing protein	1.308	1.81 × 10^−20^
*MSMEG_1275*	HNH nuclease	1.096	4.64 × 10^−10^
*MSMEG_2148*	HNH endonuclease domain-containing protein	1.081	4.62 × 10^−5^

^a.^ FC, log2 fold change.

## Data Availability

Not applicable.

## Supplementary Materials

The following supporting information can be downloaded at: https://www.mdpi.com/article/10.3390/microorganisms10081535/s1, Figure S1. Genetic organization and co-transcription analysis of *MSMEG_6762-MSMEG_6760*. Figure S2. The expression of MSMEG_6760 and MSMEG_6762 in *M. smegmatis*. Figure S3. Verification of RNA-seq results by real-time PCR. Numbers means the numbering of gene in M. smegmatis mc^2^ 155. Figure S4. SDS-PAGE gel of recombinant *M. smegmatis* protein expressed and purified from *E. coli*. Figure S5. The effect of MSMEG_6762 on *M. smegmatis* growth when overexpressed by pNIT plasmid. Table S1. Bacterial strains and plasmids in this study. Table S2. Oligonucleotides used for RT-PCR, cloning (restriction sites are underlined and in bold), site directed mutagenesis (target mutated nucleotides are underlined), knockout and verification (restriction sites are underlined), and EMSA. Table S3: Upregulated and downregulated genes upon MSMEG_6762 overexpression. References [77,78,79] are cited in the supplementary materials.

## Abbreviations

PCD	programmed cell death
TA	toxin–antitoxin
CFU	colony-forming units
dsDNA	double-stranded DNA
TUNEL assay	Terminal deoxynucleotidyl transferase dUTP nick end labeling assay
LB	Luria-Bertani
EMSA	electrophoretic mobility shift assay
