# Supplementary material for: ArsR Family Regulator MSMEG_6762 Mediates the Programmed Cell Death by Regulating the Expression of HNH Nuclease in Mycobacteria
Source: Microorganisms. 2022 Jul 29;10(8):1535. doi: 10.3390/microorganisms10081535 (PMC9416677; doi:10.3390/microorganisms10081535)
Supplement: Supplementary file 1 [file microorganisms-10-01535-s001.zip › Supplemental Information Figure S.pdf]

Supplementary Information

ArsR family regulator MSMEG_6762 mediates the programmed cell death by regulating the expression of HNH nuclease in mycobacteria

Xiangke Duan[1, 4#], Xue Huang[1#], Junqi Xu[1#], Xue Li[1], Jingjing Niu[1], Xiaoli Du[2], Xiaoyu Wang[1], Jiang Li[1], Michael Kelly[3], Jiaohan Guo[1], Ke Zhang[1], Yu Huang[1], Biao Kan[2], Jianping Xie[1*]

[1] Institute of Modern Biopharmaceuticals, State Key Laboratory Breeding Base of Eco-Environment and Bio-Resource of the Three Gorges Area, School of Life Sciences, Southwest University, Chongqing 400715, China

[2] National Institute for Communicable Disease Control and Prevention, Chinese Center for Disease Control and Prevention, Beijing 102206, China

[3] Saint John's University, 2850 Abbey Plaza, Collegeville, MN, 56321, USA

[4] Shenzhen Center for Disease Control and Prevention, Shenzhen, 518055, China

Running Head: Programmed cell death in mycobacteria

* Correspondence to Jianping Xie. Email: georgex@swu.edu.cn

# These authors contributed equally to this work

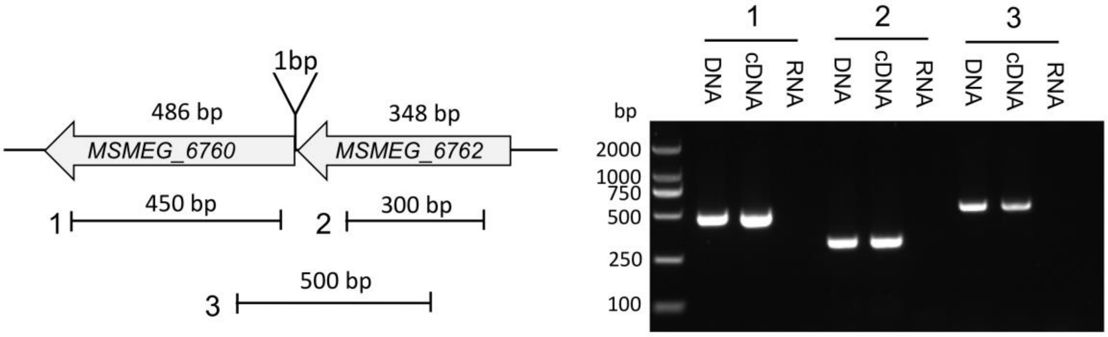

Figure S1. Genetic organization and co-transcription analysis of *MSMEG_6762-MSMEG_6760*. Total RNAs extracted from *M. smegmatis* mc^2 155 were used to synthesize cDNAs. PCR was carried out with primer pairs indicated above the lanes.

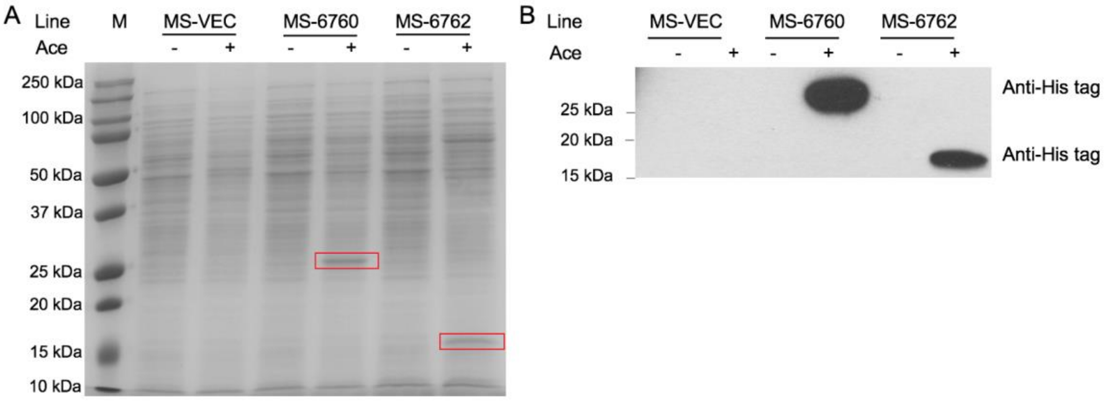

Figure S2. The expression of MSMEG_6760 and MSMEG_6762 in *M. smegmatis*. (A) The total protein of MS-VEC, MS-6760 and MS-6762 in the absence/presence inducer. (B) Western blot analysis of the expression of MSMEG_6760 and MSMEG_6762 in *M. smegmatis*.

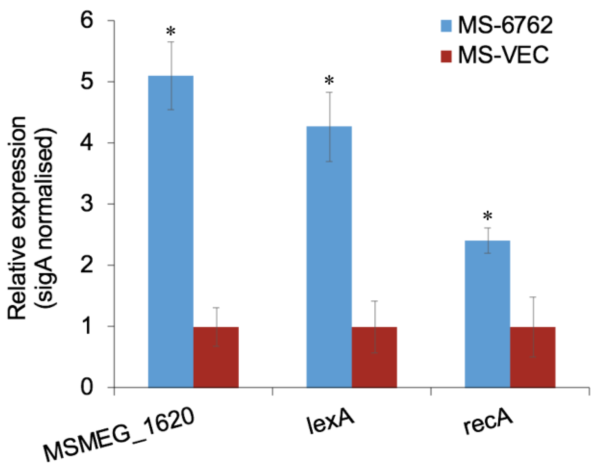

Figure S3. Verification of RNA-seq results by real-time PCR. Numbers means the numbering of gene in M. smegmatis mc^2 155. The data were averaged from three independent experiments ± s.d. Significant differences (*$p < 0.05$).

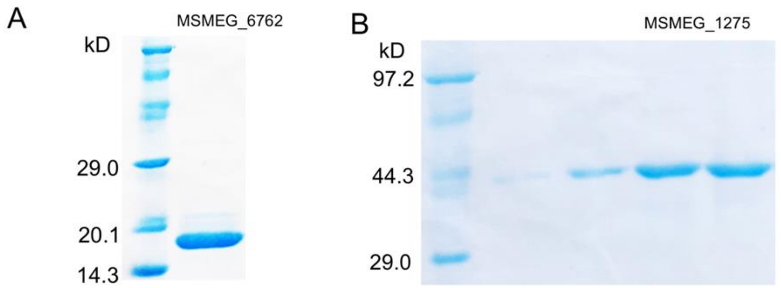

Figure S4. SDS-PAGE gel of recombinant *M. smegmatis* protein expressed and purified from *E. coli*. (A) Wild type (WT) MSMEG_6762 (18.94 kD). (B) MSMEG_1275 (45.61kD).

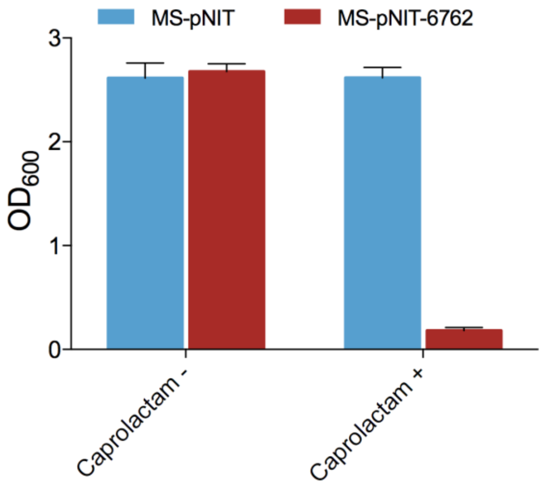

Figure S5. The effect of MSMEG_6762 on *M. smegmatis* growth when overexpressed by pNIT plasmid. OD_{600} of MS-pNIT and MS-pNIT-6762 were measured after 24 h growth with and without caprolactam (final concentration 28 mM).
