# Supplementary material for: ArsR Family Regulator MSMEG_6762 Mediates the Programmed Cell Death by Regulating the Expression of HNH Nuclease in Mycobacteria
Source: Microorganisms. 2022 Jul 29;10(8):1535. doi: 10.3390/microorganisms10081535 (PMC9416677; doi:10.3390/microorganisms10081535)
Supplement: Supplementary file 1 [file microorganisms-10-01535-s001.zip › Supplemental Information Table S1.pdf]

| | Description [a] | Source of ref. |
|---|---|---|
| Plasmids | | |
| pALACE | *E. coli–Mycobacteria* shuttle vector; Hyg $^R$ | (Du et al. 2014) |
| pNIT | *E. coli–Mycobacteria* shuttle vector; Kan $^R$ | (Pandey et al. 2009) |
| pJV53 | Expression of Che9c gp61; Kan $^R$ | (Kessel and Hatfull 2008) |
| pAL-75 | Providing hygromycin resistance cassette | (Kessel and Hatfull 2008) |
| pALACE-6762 | Expression vector for *MSMEG_6762*; Hyg $^R$ | This study |
| pALACE-5583 | Expression vector for *MSMEG_5583*; Hyg $^R$ | This study |
| pALACE-5876 | Expression vector for *MSMEG_5876*; Hyg $^R$ | This study |
| pALACE-3404 | Expression vector for *MSMEG_3404*; Hyg $^R$ | This study |
| pALACE-1275 | Expression vector for *MSMEG_1275*; Hyg $^R$ | This study |
| pALACE-2148 | Expression vector for *MSMEG_2148*; Hyg $^R$ | This study |
| *E. coli* | | |
| DH5α | supE44 Δ lac*U169*(φ 80lacZΔ M15) *hsdR17recA1 endA1 gyrA96thi-1 relA1*λ pir | Invitrogen |
| *M. smegmatis* | | |
| mc$^2$ 155 | Electrocompetent wild-type strain of *M. smegmatis* | (Snapper et al. 1990) |
| MS-VEC | mc$^2$ 155 with pALACE plasmid | This study |
| MS-6762 | mc$^2$ 155 with pALACE- *MSMEG_6762* plasmid | This study |
| MS-5583 | mc$^2$ 155 with pALACE- *MSMEG_5583* plasmid | This study |
| MS-5876 | mc$^2$ 155 with pALACE- *MSMEG_5876* plasmid | This study |
| MS-3404 | mc$^2$ 155 with pALACE- *MSMEG_3404* plasmid | This study |
| MS-1275 | mc$^2$ 155 with pALACE- *MSMEG_1275* plasmid | This study |
| MS-2148 | mc$^2$ 155 with pALACE- *MSMEG_2148* plasmid | This study |
| △*MSMEG_1275* | Derivative mc$^2$ 155 of carrying an unmarked deletion in *MSMEG_1275* | This study |
| △1275-6762 | △*MSMEG_1275* strain with pALACE-6762 plasmid | This study |
| △1275-VEC | △*MSMEG_1275* strain with pALACE plasmid | This study |

Table S1. Bacterial strains and plasmids in this study. a. The following abbreviations are used: Hyg $^R$, hygromycin B resistance; Kan $^R$, kanamycin resistance

## Reference


Du Q, Long Q, Mao J, Fu T, Duan X, Xie J (2014) Characterization of a novel mutation in the overlap of tlyA and ppnK involved in capreomycin resistance in Mycobacterium. IUBMB life 66(6):405-414

Kessel JCv, Hatfull GF (2008) Mycobacterial recombineering Chromosomal Mutagenesis. Springer, pp 203-215

Pandey AK, Raman S, Proff R, Joshi S, Kang C-M, Rubin EJ, Husson RN, Sassetti CM (2009)

Nitrile-inducible gene expression in mycobacteria. Tuberculosis 89(1):12-16

Snapper SB, Melton RE, Mustafa S, Kieser T, Jacobs WR, Jr. (1990) Isolation and characterization of efficient plasmid transformation mutants of Mycobacterium smegmatis. Mol Microbiol 4(11):1911-9
