# Supplementary material for: ArsR Family Regulator MSMEG_6762 Mediates the Programmed Cell Death by Regulating the Expression of HNH Nuclease in Mycobacteria
Source: Microorganisms. 2022 Jul 29;10(8):1535. doi: 10.3390/microorganisms10081535 (PMC9416677; doi:10.3390/microorganisms10081535)
Supplement: Supplementary file 1 [file microorganisms-10-01535-s001.zip › Supplemental Information Table S2.pdf]

Name	Forward (F) (5’-3’)[a]	Reverse (R) (5’-3’)
**Oligonucleotides used for expression vector construction**		
pALACE-*MSMEG_6762*	CGGGATCCTTGTACGTTGTGTGGGTGACCGTGA	CCATCGATATGTCAGTCCTCCTTGAGCGGTTCG
pALACE-*MSMEG_5583*	TGCCTTAAGATGTGTCTACTTGTCGTACCCGTCT	AATTCCATATGTTGCGTTGATCCGTGGCCCAATTGT
pALACE-*MSMEG_1756*	TGCCTTAAGGAGGTTGCGTTAGGCATGCCCGAAG	AATTCCATATGGCAGTCAGGACGCCGTTTGGCACA
pALACE-*MSMEG_5876*	TGCCTTAAGTCTACTGCAGCAATGTGTGCCAGGG	AATTCCATATGCGACGAGAACTAGTACGACTTGCC
pALACE-*MSMEG_3404*	TGCCTTAAGTTCTGCGATGATGGGGTCATGACGT	AATTCCATATGTCTCGTCGCTCAACCTAGTTGGCTG
pALACE-*MSMEG_1275*	TGCCTTAAGGGTGGCGTGACAGGAGCATCCGATC	AATTCCATATGCGAGCCACACTGAGAATCACCGGT
pALACE-*MSMEG_2148*	TGCCTTAAGGAGGAGCAAAGTGTGTGGTTCAGTT	AATTCCATATGCGTGAGCGCTATTTCCCTTTTGGTT
pNIT-*MSMEG_6762*	CGCATATGGAATTCATGTGGGTGACCGTGATCGACGAG	CGCATGCGGATCCGTCCTCCTTGAGCGGTTCGGATGA
**Oligonucleotides used for site directed mutagenesis**		
6762_{L18A}	GCGCTCTTCCACGCGGTAGCCGACCGCACGAGG	CCTCGTGCGGTCGGCTACCGCGTGGAAGAGCGC
6762_{R24A}	CTCGCCGACCGCACGGTACGCGACATCATGCGC	GCGCATGATGTCGCGTACCGTGCGGTCGGCGAG
6762_{H54A}	GCGGCGGTGCAGAAGGTAGTTGCGGTGCTGGAG	CTCCAGCACCGCAACTACCTTCTGCACCGCCGC
6762_{L58A}	GAAGCATGTTGCGGTGGTAGAGAAAGCCGGCCTG	GCAGGCCGGCTTTCTCTACCACCGCAACATGCTTC
1275_{H258A}	GAACAACCCGATCGACGTAGTGCTGCCCTGGTCAC	GTGACCAGGGCAGCACTACGTCGATCGGGTTGTTC
1275_{N272A}	TATCGACGGCCTGGCCGTACTGGTGCTGGCGTGCG	CGCACGCCAGCACCAGTACGGCCAGGCCGTCGATA
1275_{N281A}	GGCGTGCGCGCGCTGCGTAGGCGACAAGGGTGGC	CGCCACCCTTGTCGCCTACGCAGCGCGCGCACGCC
**Oligonucleotides used for knockout and verification**		
KO1275-1	CGGGATCCGCGAACTTCTTCAGCGACAGGTCAT	AGATCTGCCACCCCCATGTCGGTGCACTGCG
KO1275-2	TGCACCGACATGGGGGTGGCAGATCTTTCTCAGTGTGGCTCGAAACCCG	GGGGTACCGATTTCACCACGATGTCGTTGGGCG
1275veri	CGCTGTCGGTTCCCTTCGTGATAAT	TGTACATCGACTCTCTTCTCAGCCC
**Cloning and protein purification**		
pET6762	CGGAATTCGTTGTGTGGGTGACCGTGATCGACG	CCCAAGCTTTCAGTCCTCCTTGAGCGGTTCGGAT

pET1275	CGGAATTCGTGACAGGAGCATCCGATCCGCTGCTA	ATAAGAATGCGGCCGCTACGGGTTTCGAGCCACAC
**Oligonucleotides used for EMSA**		
*MSMEG*_1275p1	GGACGCCATGTTGGATGCCATTGCC	CACGAAGGGAACCGACAGCGTGGCC
*MSMEG*_1275p2	GATGCCATTGCCAGAAGCGCTCTCG	GACGAGTACTGGGCCGCTCTGGACG
*MSMEG*_1275p3	GTCGGACAGCGTGCGGATCACCGGC	GGAACCGACAGCGTGGCCCAACTTG

Table S2. Oligonucleotides used for RT-PCR, cloning (restriction sites are underlined and in bold), site directed mutagenesis (target mutated nucleotides are underlined), knockout and verification (restriction sites are underlined), and EMSA.
